# An Additively Manufactured Sample Holder to Measure the Controlled Release of Vancomycin from Collagen Laminates

**DOI:** 10.3390/biomedicines9111668

**Published:** 2021-11-11

**Authors:** Michelle Fiona Kilb, Yannik Moos, Stefanie Eckes, Joy Braun, Ulrike Ritz, Daniela Nickel, Katja Schmitz

**Affiliations:** 1 Clemens-Schöpf-Institute of Organic Chemistry and Biochemistry, Technical University of Darmstadt, Alarich-Weiss-Straße 8, 64287 Darmstadt, Germany; michelle_fiona.kilb@tu-darmstadt.de (M.F.K.); stefanie.eckes@gmx.de (S.E.); 2 Akademische Motorsportgruppe Darmstadt e.V., c/o Institut für Verbrennungskraftmaschinen und Fahrzeugantriebe, Otto-Berndt-Straße 2, 64287 Darmstadt, Germany; yannik.moos@amda-racing.de; 3 Department of Orthopaedics and Traumatology, BiomaTiCS, University Medical Center, Johannes Gutenberg University, Langenbeckstraße 1, 55131 Mainz, Germany; joybraun@uni-mainz.de (J.B.); ritz@uni-mainz.de (U.R.); 4 Berufsakademie Sachsen–Staatliche Studienakademie Glauchau, University of Cooperative Education, Kopernikusstraße 51, 08371 Glauchau, Germany; daniela.nickel@ba-sachsen.de

**Keywords:** collagen, controlled release, crosslinking, vancomycin, additively manufactured sample holder

## Abstract

The controlled release of antibiotics prevents the spread of pathogens and thereby improves healing processes in regenerative medicine. However, high concentrations may interfere with healing processes. It is therefore advantageous to use biodegradable materials for a controlled release. In particular, multilayer materials enable differential release at different surfaces. For this purpose, collagen sheets of different properties can be bonded by photochemical crosslinking. Here, we present the development and application of an easily accessible, additively manufactured sample holder to study the controlled release of vancomycin from modularly assembled collagen laminates in two directions. As proof-of-concept, we show that laminates of collagen sheets covalently linked by rose bengal and green light crosslinking (RGX) can be tightly inserted into the device without leakage from the upper to lower cavity. We used this sample holder to detect the release of vancomycin from symmetrically and asymmetrically loaded two-layer and three-layer collagen laminates into the upper and lower cavity of the sample holder. We show that these collagen laminates are characterized by a collagen type-dependent vancomycin release, enabling the control of antibiotic release profiles as well as the direction of antibiotic release.

## 1. Introduction

The microbial contamination of a surgical wound leads to surgical site infections (SSIs), which require additional treatment and lead to prolonged recovery times [1,2]. To prevent infections, antibiotics are mostly applied systemically [3]. As this may lead to side effects or drug resistance, the local delivery of antibiotics to the surgical site is advantageous [3]. Direct application of antibiotics is effective [4]. However, since a high antibiotic concentration might be toxic for cells [5], the controlled release of antibiotics is preferable. Different delivery systems for controlled antibiotic release have been described in literature, such as nanobioceramics [6], nanoparticles as scaffolds or injections [7], polycaprolactone coated chitin-lignin gels [3] or gentamicin-collagen implants [8]. To prevent the bacterial infection of surgical wounds, different antibiotics such as gentamicin [9], tetracyclines, vancomycin, and tobramycin can be applied [10].

The glycopeptide vancomycin is used as an effective antibiotic against gram-positive bacteria, such as *Staphylococcus aureus* [11]. Since Staphylococci are the main pathogens that cause surgical site infections, vancomycin is frequently used to prevent the infection of surgical wounds [12,13]. One advantage of vancomycin is its compatibility for administration to patients with a β-lactam allergy [13]. Lopez et al. showed that different biocompatible layered double hydroxides as complexes with vancomycin are able to release vancomycin [14]. Silk fibroin nanoparticles containing vancomycin in silk scaffolds are also able to deliver vancomycin, as described by Hassani Besheli et al. [15]. Vancomycin has also been used to impregnate collagen/hydroxyapatite layers coated on titanium implants to prevent infections and improve implant integration into the bone [16]. Hartinger et al. showed that natural biomaterials such as collagen, which was crosslinked by carbodiimide, can also be used for the controlled release of vancomycin [17].

As a natural biomaterial, collagen is commonly used in regenerative medicine as it is biocompatible and is degraded to non-toxic peptides and amino acids [18,19,20,21]. Its properties can be modified by crosslinking, e.g., by glutardialdehyde [22] or carbodiimide [17,23], physical crosslinking by UV-irradiation or dehydrothermal treatment [24], or photochemical crosslinking, for which rose bengal (RB) and green light crosslinking (RGX) is a prominent example [25]. RGX-modified collagen sheets have already been used for the controlled release of vancomycin [25]. These sheets can be combined to laminates by soaking the sheets with rose bengal solution and exposing stacks of soaked collagen sheets and with intermediate layers of rose bengal solution to green light. Consequently, the combination of collagen sheets within collagen laminates can be used to tailor their properties for a given application [25]. In particular, using laminates in surgical wounds can enable the directional release of antibiotics and ensure that antibiotics reach sufficient concentrations at the infectious site [26] without interfering with bone healing [5]. Biomaterials with a layered structure have already been described by Bracaglia et al. [27], Campbell et al. [28], Drohan et al. [29], Shah et al. [30], and Wen et al. [31]. While these materials are built up in a layer-by-layer fashion, collagen sheets of different properties and different antibiotic loads can be quickly assembled and crosslinked by RGX. For this purpose, dried collagen sheets are soaked with a solution of 0.01% rose bengal and the desired amount of antibiotic, stacked in the appropriate order, and irradiated with green light, e.g., with a power LED for only 10 min [25]. RGX has been approved for several clinical applications [32,33,34] and the required irradiation apparatus is inexpensive and sufficiently small to fit into any sample preparation lab on-site.

To demonstrate that a directional release of vancomycin can be accomplished with the described collagen laminate, an appropriate sample holder is required. To our knowledge, the directionality of antibiotic release from biomaterials with a layered structure has not been quantified. Here, we describe the development of a customized additively manufactured sample holder to study the directed release of vancomycin from two-layered and three-layered collagen laminates. We considered the Boyden chamber, which is widely used for cell migration analysis [35], as an appropriate starting point for the development of a sample holder to quantify directional release. In the Boyden chamber, a microporous membrane separates a lower chamber from an upper chamber [35]. In analogy, we fabricated a sample holder that can accommodate a customized collagen laminate in a cavity of a 24-well plate, resulting in two chambers separated by the semipermeable collagen laminate. With this assembly, the release of vancomycin from the collagen laminate into the upper and lower chamber can be analyzed.

## 2. Materials and Methods

### 2.1. Fabrication of the Sample Holder

The upper and lower part of the sample holder were constructed with FreeCAD (version 0.19 Build 24276 (Git), open-source software (available from: http://www.freecadweb.org, accessed on 15 May 2021). The precise dimensions of both parts are shown in Figures S1 and S2.

As a preparation for additive manufacturing, both parts were sliced with the slicing software PreForm (version 3.14.0, Formlabs, Somerville, MA, USA). Slicing was performed with a layer thickness of 50 µm, a full raft type, a density of 1.0, and a touch point size of 0.4 mm. The support structure was automatically generated and the internal support structure was turned off.

Additive manufacturing of both parts was carried out with a Formlabs Form 3 (Formlabs, Somerville, MA, USA) with a construction volume of 14.5 × 14.5 × 18.5 cm, a layer thickness of 25–300 µm (vertical resolution), a XY-resolution of 25 µm and a laser spot size of 85 µm. The upper and the lower part of the sample holder were either fabricated with a Formlabs Clear Resin (Formlabs, Somerville, MA, USA) or a Formlabs Standard White Resin V4 (Formlabs, Somerville, MA, USA).

For postprocessing the finished sample holders were washed in isopropanol for 20 min. The sample holders were separated from the build plate and the support structures were removed from each part. Finally, the sample holders were hardened for 10 min at 60 °C in a UV light chamber.

### 2.2. Fabrication of Collagen Laminates

Collagen laminates were prepared from two different types of commercially available collagen sheets, which have been characterized by Eckes et al. [25]. The non-perforated collagen film was obtained from Collagen Solutions (London, UK) and is referred to as “Collagen Solutions”. The second collagen type, Atelocollagen sponge (CLS-01), was purchased from Koken Co. Ltd. (Tokyo, Japan) and is referred to as “Atelocollagen”. The collagen sheets were cut to squares (1 cm × 1 cm) and loaded with 0.01% (*w*/*v*) RB (Alfa Aesar, Haverhill, MA, USA) in PBS (137 mM sodium chloride (Carl Roth GmbH + Co. KG, Karlsruhe, Germany), 2.7 mM potassium chloride (Carl Roth GmbH + Co. KG, Karlsruhe, Germany), 1.5 mM potassium dihydrogen phosphate (Carl Roth GmbH + Co. KG, Karlsruhe, Germany) and 8.1 mM disodium hydrogen phosphate dihydrate (Carl Roth GmbH + Co. KG, Karlsruhe, Germany), pH 7.4) with or without 1000 µg of vancomycin hydrochloride (Carl Roth GmbH + Co. KG, Karlsruhe, Germany), by swelling the sheets in a Petri dish (SARSTEDT, Nümbrecht, Germany) for 2 h. Loading was performed in the dark with a volume that corresponds to the loading capacity with PBS, as described by Eckes et al. [25]. In brief, the loading volume was determined after incubating the sheets in PBS for 2 h at room temperature (RT). After loading, the collagen laminates were prepared. For this purpose, 20 µL of 0.01% (*w*/*v*) RB in PBS were spread onto one sheet and the second sheet was placed onto the first one (two-layer laminates). For three-layer laminates, additional 20 µL of 0.01% (*w*/*v*) RB in PBS were spread onto the second sheet and the third sheet was placed onto the second one. The stapled sheets were exposed to green light (λ = 565 nm) for 10 min using a mounted LED (M565L3, Thorlabs GmbH, Bergkirchen, Germany) to crosslink the sheets with each other, as described by Eckes et al. [25].

### 2.3. Proof of Tightness

To analyze the tightness of the sample holder, a two-layer collagen laminate consisting of Collagen Solutions and Atelocollagen without vancomycin was prepared and placed in the sample holder which was then inserted into a cavity of a non-treated 24-well tissue culture plate (VWR International, Radnor, PA, USA) filled with 1 mL of PBS (lower cavity). In the next step, 1 mL of 0.005% (72.3 µM) bromophenol blue sodium salt (Serva Electrophoresis GmbH, Heidelberg, Germany) in PBS was filled into the upper part of the sample holder to cover the collagen laminate (upper cavity). 120 µL samples were taken from the upper and lower cavity at the beginning and after 1 h, 2 h and 24 h of incubation at RT. The absorption of bromophenol blue at 595 nm was measured in triplicates (3 × 30 µL) in a clear polystyrene flat bottom non-sterile 384 well microplate (Corning Inc., Corning, NY, USA) in a microplate reader (Infinite M-1000, Tecan Group AG, Männedorf, Switzerland). The concentration of bromophenol blue in each sample was determined by a calibration series of bromophenol blue sodium salt in PBS (72.3 µM to 0.28 µM) which was freshly prepared for each experiment.

### 2.4. Release of Vancomycin

Immediately after preparing the collagen laminates, the samples were placed into the lower part of the sample holder by using tweezers. Next, the upper part of the sample holder was pushed into the lower part fixing the collagen laminate between both parts. The sample holder was placed in a well of a non-treated 24-well sterilized tissue culture plate (VWR International, Radnor, PA, USA), which was filled with 1 mL of PBS (lower cavity) and 1 mL of PBS was filled into the upper part of the sample holder to cover the collagen laminate (upper cavity). The tissue culture plate was incubated at 37 °C and samples from the upper and lower cavity were taken at different time points. For this purpose, the liquid was completely withdrawn and 1 mL of fresh PBS was added to both, the upper and lower cavity. Sample analysis was performed with three replicates by reversed-phase HPLC with a C18 Synergi^TM^ 4 µm Fusion-RP 80 Å 250 × 4.6 mm column (Phenomenex Inc., Torrance, CA, USA) as described by Eckes et al. [25]. In brief, 100 µL of the sample were analyzed with gradient elution by measuring the absorption at 280 nm.

## 3. Results

### 3.1. Development of an Additively Manufactured Sample Holder for Release Experiments

**Figure 1:**
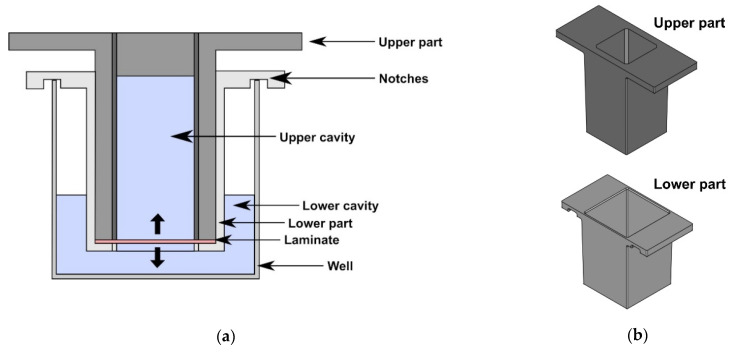
Illustration of the sample holder and its two parts. (**a**) Cross section of the sample holder inserted into the cavity of a 24-well plate. The sample holder consists of an upper and a lower part that are used to position the laminate. If the laminate-loaded sample holder is placed into the cavity of the 24-well plate, an upper and a lower cavity are formed, allowing an analysis of vancomycin release into both directions (thick arrows). (**b**) Isometric view of the upper part and the lower part. The construction drawing was created with FreeCAD (version 0.19).

The sample holder for the analysis of the controlled release of vancomycin from multilayered collagen biomaterials needs to meet the following requirements: To allow the quantification of antibiotic release in opposing directions, the multilayer collagen laminate should separate two cavities from each other with a filling volume of 1 mL per cavity. Furthermore, the sample holder with the collagen laminate should fit into a well of a conventional 24-well tissue culture plate. Finally, the collagen laminate needs to be tightly inserted, to avoid any fluid leakage from one chamber to the other. As an easily accessible fabrication technique that allows rapid prototyping and adjustments to an existing layout, additive manufacturing was chosen for the fabrication of the sample holder. Figure 1 shows an illustration of the sample holder and its two parts.

During the development of the sample holder, additional features were introduced and the dimensions were adjusted. The best spacing for the collagen laminate between both parts of the sample holder was determined to a value of 0.2 mm and both the walls of the lower part and the outer walls of the upper part were tilted to wedge the upper part into the lower to ensure tightness of the sample holder.

The sample holder was further improved in an iterative process. The laminate supporting surface of the lower part was adjusted and its edges were phased to enhance the positioning of the laminate. Furthermore, notches were added to the wings of the lower part to enhance the positioning of the sample holder in the well. The dimensions of the sample holder can be found in Figures S1 and S2.

### 3.2. Proof of Tightness

**Figure 2:**
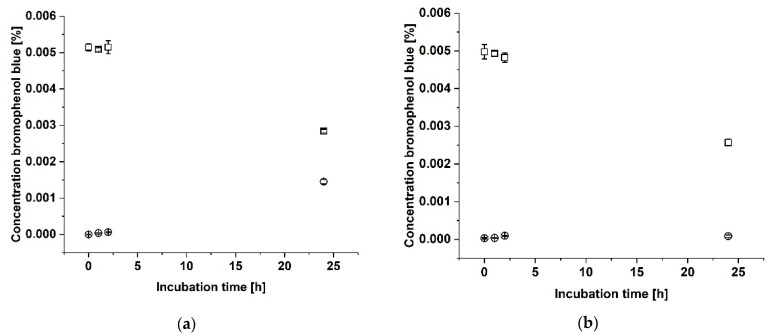
Proof of tightness of the sample holder. The sample holder was fabricated by additive manufacturing from a standard transparent resin. A two-layer collagen laminate consisting of Collagen Solutions and Atelocollagen was placed in the sample holder. The tightness of the sample holder was demonstrated by analyzing the diffusion of bromophenol blue from the upper cavity (squares) into the lower cavity (dots). (**a**) Concentration of bromophenol blue in a setup with a collagen laminate. (**b**) Concentration of bromophenol blue in a setup with a collagen laminate supported by a layer of parafilm.

The first version of the sample holder was fabricated with a Formlabs Clear Resin. The tightness of the sample holder was demonstrated by analyzing the diffusion of bromophenol blue from the upper cavity into the lower cavity with both cavities separated by a two-layered collagen laminate, consisting of Collagen Solutions and Atelocollagen. As shown in Figure 2a, the bromophenol blue concentration in the upper cavity did not change during the first 2 h of incubation but decreased after 24 h. Correspondingly, the bromophenol blue concentration in the lower cavity (see Figure 2a) increased after 24 h of incubation. To decide whether the diffusion of bromophenol blue from the upper into the lower cavity can be attributed to the permeability of the laminate or to a leakage of the sample holder, the experiment was repeated with a layer of parafilm located underneath the two-layered collagen laminate as a non-permeable layer. As depicted in Figure 2b, the bromophenol blue concentration in the upper cavity decreased after 24 h but did not increase in the lower cavity after 24 h (see Figure 2b). A blue discoloration of the laminate was observable. Since the non-permeable laminate did not show any increase of bromophenol blue in the lower cavity after 24 h compared to the laminate, the sample holder was considered as tight. For further experiments, the sample holder was fabricated from a standard white resin that contributed to a better handling.

### 3.3. Release of Vancomycin

**Figure 3:**
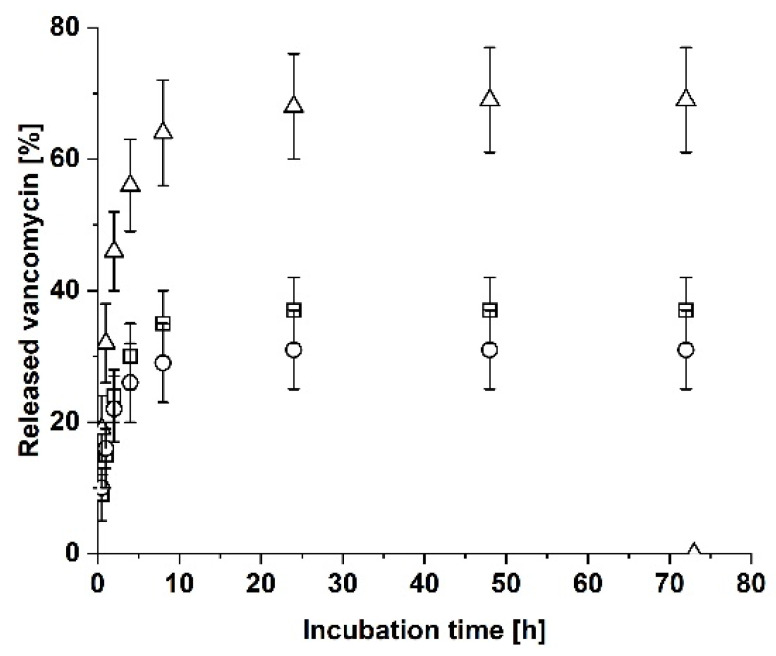
Release of vancomycin from a sheet of RGX-modified Atelocollagen that was placed in the sample holder. Atelocollagen was loaded with vancomycin and the release in the upper cavity (squares), the lower cavity (dots) and in total (triangles) was analyzed. After 72 h, the collagen sheet was incubated for an additional hour in PBS without the sample holder. The error bars represent the standard deviation (*n* = 3).

The total release of vancomycin from a single RGX-modified sheet of Atelocollagen in solution has already been analyzed by Eckes et al., who observed a half-maximal release of vancomycin within 0.5 h [25]. To quantify whether the sample holder has an effect on the release rate of vancomycin, the release of vancomycin from a single RGX-modified Atelocollagen sheet placed in the sample holder was analyzed. After 72 h, the laminate was incubated with 1 mL PBS without the sample holder for an additional hour to test whether the sample holder retains any amount of vancomycin. As displayed in Figure 3, the total amount of released vancomycin reached a constant level after 24 h (24 h: 68 ± 8%, 48 h: 69 ± 8%, 72 h: 69 ± 8%). The total half-maximal release was reached within 1 h (32 ± 6%), which is 30 min later than for the single sheet in solution [25]. The upper and the lower cavity did not show any differences regarding the released amounts of vancomycin. An additional hour of incubation in solution without the sample holder did not lead to an additional release of vancomycin. To test whether the insertion of the sample in the sample holder might lead to the loss of vancomycin, RGX-modified, vancomycin-loaded Atelocollagen was first inserted into the sample holder and then removed and placed into a cavity filled with 1 mL of PBS and incubated for 24 h at 37 °C. For a comparison, a second sample was directly incubated under the same conditions. The percentage of vancomycin released from the sample in the sample holder and from the directly incubated sample showed no significant difference (sample holder: 53 ± 1%, directly incubated: 50 ± 1%).

**Figure 4:**
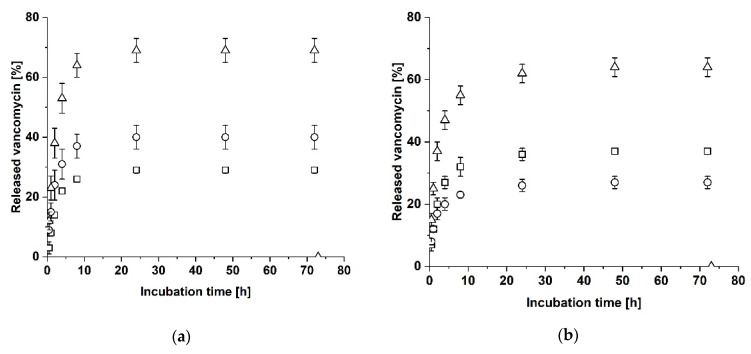
Release of vancomycin from a two-layer laminate consisting of Atelocollagen and Collagen Solutions. Atelocollagen was loaded with vancomycin and the release in the upper cavity (squares), the lower cavity (dots) and in total (triangles) was analyzed. After 72 h, the collagen sheet was incubated for an additional hour in PBS without the sample holder. The error bars represent the standard deviation (*n* = 3). (**a**) Atelocollagen facing the upper cavity. (**b**) Atelocollagen facing the lower cavity.

In the next step, the release of vancomycin from a two-layer laminate consisting of Collagen Solutions and Atelocollagen was analyzed. For this purpose, we loaded Atelocollagen with vancomycin and placed the laminate in the sample holder, with Atelocollagen facing the upper cavity. The release of vancomycin was observed over 72 h. After 72 h, the laminate was incubated with 1 mL PBS without the sample holder for an additional hour. As shown in Figure 4a, the release of vancomycin reached a maximal total value of 69 ± 4% after 24 h and did not change after 48 h or 72 h of incubation. The total half-maximal release was reached within 2 h (38 ± 5%), which is 1 h later than the single RGX-modified sheet of Atelocollagen in the sample holder. Interestingly, more vancomycin was released into the lower cavity than into the upper cavity, even though the side of the laminate loaded with vancomycin faced the upper cavity. As in the previous experiment, an additional hour of incubation in solution without the sample holder did not lead to an additional release of vancomycin. To analyze whether the position of the laminate in the sample holder has an influence on the release of vancomycin, we repeated the experiment with the two-layer laminate with Atelocollagen loaded with vancomycin but facing the lower cavity. As before, the amount of released vancomycin reached a constant level after 24 h (24 h: 62 ± 3%, 48 h: 64 ± 3%, 72 h: 64 ± 3%) and the total half-maximal release was reached within 2 h (37 ± 3%, see Figure 4b). No further release was detected upon incubation for one hour in solution. This time, more vancomycin was released into the upper cavity indicating that vancomycin was preferentially released at the side with Collagen Solutions even though it had been loaded into the Atelocollagen layer.

**Figure 5:**
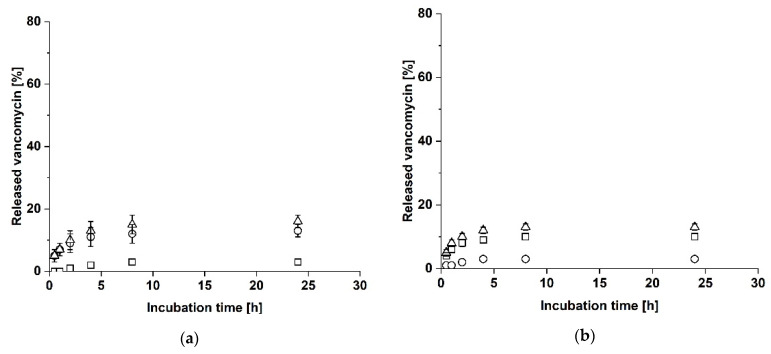
Release of vancomycin from a two-layer laminate consisting of Atelocollagen and Collagen Solutions. Collagen Solutions was loaded with vancomycin and the release in the upper cavity (squares), the lower cavity (dots) and in total (triangles) was analyzed. The error bars represent the standard deviation (*n* = 3). (**a**) Collagen Solutions facing the lower cavity. (**b**) Collagen Solutions facing the upper cavity.

Since the measured release reached a constant level after 24 h and no additional release of vancomycin was detectable after an additional hour of incubation in solution, the release of vancomycin was analyzed only over 24 h in subsequent experiments. To verify, if the asymmetrical release from the laminate could be explained by the structural properties of crosslinked Atelocollagen, the experiment was repeated with the two-layer laminate with vancomycin loaded in the Collagen Solutions sheet, either facing the upper or lower cavity. Positioning the laminate with loaded Collagen Solutions facing the lower cavity resulted in a total maximal release of 16 ± 2% after 24 h (see Figure 5a) which is less than the release from Atelocollagen in the two-layer laminate. The total half-maximal release was reached within 1 h (7 ± 2%), which is one hour earlier than the half-maximal release from Atelocollagen in the two-layer laminate. The lower cavity showed a higher release of vancomycin than the upper cavity. Positioning the laminate with loaded Collagen Solutions facing the upper cavity resulted in a total maximal release of 13 ± 1% after 24 h (see Figure 5b) similar to the previous result. Likewise, the total half-maximal release was reached within 1 h (8 ± 1%). This time, the upper cavity showed an increased release of vancomycin compared to the lower cavity, so that in both experiments, more vancomycin was released into the cavity facing the loaded Collagen Solutions sheet.

**Figure 6:**
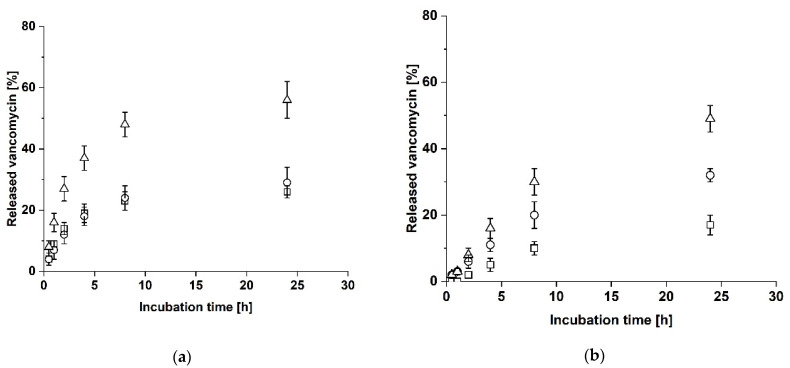
Release of vancomycin from symmetrical three-layer laminates. The release in the upper cavity (squares), the lower cavity (dots) and in total (triangles) was analyzed. The error bars represent the standard deviation (*n* = 3). (**a**) Atelocollagen loaded with vancomycin between two sheets of Collagen Solutions (laminate CAC). (**b**) Atelocollagen loaded with vancomycin between two sheets of Atelocollagen (laminate AAA).

In the next step the release of vancomycin from symmetrical three-layer laminates was analyzed (see Figure 6). As higher total amounts had been released from the Atelocollagen sheet in the two-layer laminate than from the Collagen Solutions sheet, vancomycin was loaded to a central Atelocollagen sheet in three-layer laminates. The three-layer laminate CAC with Collagen Solutions as outer layers reached a total maximal release of 56 ± 6% of vancomycin after 24 h of incubation at 37 °C (see Figure 6a). The total half-maximal release was reached within 2 h (27 ± 4%), which is comparable to the time for half-maximal release of the two-layer laminate with the loaded Atelocollagen sheet. In contrast to the two-layer laminate, the same amount of vancomycin was released into both cavities. The three-layer laminate AAA with the central layer loaded with vancomycin showed a total maximal release of 49 ± 4% vancomycin after 24 h of incubation at 37 °C (see Figure 6b). In contrast to the CAC laminate, the time of half-maximal release was extended to 8 h (30 ± 4%) and more vancomycin was released into the lower cavity than to the upper cavity. We also observed a swelling of the bottom side of the laminate into the lower cavity.

## 4. Discussion

### 4.1. Proof of Tightness

Tightness analysis with bromophenol blue showed that the dye from the upper cavity was detectable in the lower cavity after 24 h when both cavities were separated by a two-layered collagen laminate. This might be explained by the permeability of the collagen laminate to bromophenol blue. Therefore, the experiment was repeated with an additional layer of parafilm as a non-permeable material facing the lower chamber. This resulted only in a decrease of the bromophenol blue concentration in the upper cavity, but not in an increase in the lower cavity after 24 h. The upper side of the laminate was colored blue after 24 h, suggesting that the laminate had absorbed the bromophenol blue, which explains the decrease of the bromophenol blue concentration in the upper cavity. Since the parafilm layer underneath the laminate prevented leakage through the laminate itself, no bromophenol blue reached the lower cavity. In conclusion these experiments show that collagen laminates are permeable to small molecules and that the sample holder is sufficiently tight to prevent leakage around the sides of the laminate.

### 4.2. Release of Vancomycin

As described earlier, the proportion of directionally released vancomycin is of interest during antibiotic treatment since antibiotics have to reach sufficient concentrations at the infectious site [26]. For this purpose, we analyzed the release of vancomycin from modularly assembled collagen laminates in two directions. An overview of the results from the vancomycin release experiments is shown in Table 1. First, the release of vancomycin from a single RGX-modified sheet of Atelocollagen placed in the sample holder was analyzed, to quantify whether the sample holder has an effect on the amount and time of vancomycin release. Our results showed that the total amount of released vancomycin within 72 h did not reach 100%. As an additional hour of incubation in solution did not show any further release of vancomycin, the sample holder should not influence the total amount of released vancomycin. Furthermore, the incomplete release of vancomycin from crosslinked collagen has already been described in literature [17,25]. However, the effect of compression caused by the sample holder as well as the re-swelling of hydrogels after compression should not be neglected. Hadjipanayi et al. describe that the compression of collagen gels leads to a cumulative loss of fluid from the collagen gel, especially during the first minutes of compression [36]. Consequently, the incomplete release of vancomycin might also be attributed to a loss of vancomycin-containing fluid upon inserting the collagen sample into the sample holder. However, as the comparison of the sample directly incubated or inserted into the sample holder before incubation only led to a release difference of 1%, the compression caused by the sample holder does not explain the incomplete release of vancomycin.

Our data showed that the total half-maximal release was reached 30 min later compared to a single sheet in solution [25], which suggests that the sample holder prolongs the release time of vancomycin. This might be explained by the fact that the contact areas of the upper and lower part of the sample holder decrease the release area by about 37% from 1 cm^2^ to 0.635 cm^2^. Furthermore, the upper and the lower cavity did not show any differences regarding the released amounts of vancomycin for a single sheet, which meets the expectations as both release areas are equivalent.

The release of vancomycin from a two-layer laminate with vancomycin loaded in Atelocollagen facing the upper cavity led to similar values for the total maximal release, however, the total half-maximal release was reached one hour later than with the single sheet of Atelocollagen in the sample holder. Moreover, when the vancomycin-loaded Atelocollagen layer faced the lower cavity, an increased release into the upper cavity was observed, so that the structure of the crosslinked Atelocollagen-Collagen Solutions laminate seems to be responsible for the asymmetrical release of vancomycin. In particular, the physical properties of both collagen materials differ from each other in that crosslinked Atelocollagen showed a higher swelling degree than crosslinked Collagen Solutions [25]. This might indicate a higher porosity of Atelocollagen compared to Collagen Solutions [25]. The porous structure of Atelocollagen might be compressed during insertion into the sample holder. Kihara et al. showed that the diffusion coefficient of biomolecules with a comparable size to vancomycin decreased in condensed collaged gels compared to biomolecules in solution or biomolecules in non-condensed collagen gels [37]. The decrease of diffusivity by compression has also been described by Leddy et al. [38]. As compressed hydrogels re-swell after being re-exposed to solution [39], the fluid uptake during re-swelling might slow down the release of vancomycin. If this assumption is true, then this effect should be also observed if vancomycin is loaded into a Collagen Solutions sheet with its more compact structure, as the structural properties of Atelocollagen reduce the release of vancomycin at the Atelocollagen surface. To verify this hypothesis, the experiment was repeated with the two-layer laminate with vancomycin loaded in the Collagen Solutions sheet, either facing the upper or lower cavity. Our data confirm this assumption, as loaded Collagen Solutions facing the upper cavity releases more vancomycin into the upper cavity than into the lower cavity and vice versa. However, the total release from this assembly was significantly lower than the release from Atelocollagen and AC-laminate with vancomycin-loaded Atelocollagen. This might be explained by the different structure and the effect of RGX, as crosslinking leads to an increased stability of Atelocollagen [25]. As already described by Eckes et al., Atelocollagen is thicker and more porous than the tightly packed Collagen Solutions, resulting in longer diffusion paths [25]. For the thinner and more compact Collagen Solutions, the compressive effect and the effect of fluid uptake during re-swelling in the opposite direction of antibiotic release should hardly influence the vancomycin release. Consequently, the half-maximal release from Collagen Solutions is expected to be earlier than from Atelocollagen, as the data show. The observed compressive effect is of practical relevance as the material will be compressed if applied to a wound.

The vancomycin release from the symmetrical three-layer laminate CAC resulted in similar maximal values for the total release as well as a similar period for half-maximal release as the two-layer laminates with loaded Atelocollagen. This meets the expectations as Collagen Solutions is very thin [25] and should therefore not have any additional effect on the release time. However, the additional layer of Collagen Solutions resulted in an equal release of vancomycin into the upper and lower cavity as opposed to the asymmetric two-layer laminate with loaded Atelocollagen. This was to be expected as the symmetrical laminate (CAC) has release areas with identical surface area and material properties. The additional layer of Collagen Solutions can therefore counter the influence of Atelocollagen on the release of vancomycin. In contrast, the time for total half-maximal release from the symmetrical laminate AAA was four times longer than for CAC, even though the total maximal release reached a similar value as with CAC. As described for the two-layer laminates, this might be explained by the material properties of the thick and porous Atelocollagen layers on the release of vancomycin. Most interestingly, we observed an unequal release of vancomycin for the symmetrical laminate AAA. The observed swelling of the bottom side of the laminate into the lower cavity indicates that the bottom and top side of the laminate cannot be considered as equal due to the increased release area at the bottom side. This might explain the release of more vancomycin into the lower cavity than into the upper cavity. The swelling of AAA at the bottom side of the sample holder might be explained by the thickness of AAA, leading to a force effect from top to down upon closure of the sample holder. Since the asymmetrical swelling does not occur with CAC, the compact Collagen Solutions might have a stabilizing effect on the central Atelocollagen layer. In future experiments, a lattice structure at the bottom of the lower and upper part of the sample holder might simplify the positioning of rather flexible biomaterials and allow for more even forced distribution upon sample insertion. This way, the sample holder can be easily customized to the properties of the respective biomaterial (e.g., its size, flexibility, and swelling degree).

## 5. Conclusions

Modularly assembled laminates of collagen sheets of different texture and loading enable the controlled release of antibiotically active compounds. They can be produced in an inexpensive procedure on-site to meet the needs for customized surgical wound care. The developed additively manufactured sample holder enables a simple quantification of directional vancomycin release from modularly assembled collagen laminates. It is a convenient and easily accessible tool to study the directional release from biomaterials. Asymmetric AC collagen laminates show a preferential release of vancomycin on the side with Collagen Solutions. The half-maximal release is reached one hour earlier if vancomycin is loaded into Collagen Solutions compared to loading into Atelocollagen. The differences between both release directions in terms of the percentage of released vancomycin after 24 h lie between 16% and 63%. These results enable the control of antibiotic release profiles as well as the direction of antibiotic release. The thicker, more porous Atelocollagen leads to prolonged release times due to longer diffusion paths and re-swelling after compression during handling whereas the thin and more compact Collagen Solutions provides short diffusion paths and can stabilize the softer Atelocollagen.

## 6. Patents

Schmitz, K.; Ritz, U.; Nickel, D.; Oechsner, M.; Beyrich, T.; Rommens, P.M.“ Antimikrobielle, gewebsregenerierende Laminate für die regenerative Medizin” (DE102017126149_A1).

## Figures and Tables

**Table 1:** Overview of the results from vancomycin release experiments. The name of each laminate or single collagen sheet refers to its composition, as the laminates are composed of single sheets of Atelocollagen (A) and/or Collagen Solutions (C).

Laminate or Single Collagen Sheet		Amount of Released Vancomycin		Additional Information
Name	Property	After 24 h	Half-Maximal Release	
A	RGX-modified	68 ± 8%	32 ± 6% (1 h)	Equal release, no additional release after incubation without sample holder
AC	Vancomycin-loaded A faces upper cavity	69 ± 4%	38 ± 5% (2 h)	Unequal release (C > A), no additional release after incubation without sample holder
AC	Vancomycin-loaded A faces lower cavity	62 ± 3%	37 ± 3% (2 h)	Unequal release (C > A), no additional release after incubation without sample holder
AC	Vancomycin-loaded C faces lower cavity	16 ± 2%	7 ± 2% (1 h)	Unequal release (C > A)
AC	Vancomycin-loaded C faces upper cavity	13 ± 1%	8 ± 1% (1 h)	Unequal release (C > A)
CAC	Vancomycin-loaded A as central layer	56 ± 6%	27 ± 4% (2 h)	Equal release
AAA	Vancomycin-loaded A as central layer	49 ± 4%	30 ± 4% (8 h)	Unequal release (lower cavity > upper cavity)

## Data Availability

The data presented in this study are available on request from the corresponding author.

## Supplementary Materials

The following are available online at https://www.mdpi.com/article/10.3390/biomedicines9111668/s1. Figure S1: Dimensions of the lower part of the sample holder; Figure S2: Dimensions of the upper part of the sample holder.
