# Supplementary material for: An Additively Manufactured Sample Holder to Measure the Controlled Release of Vancomycin from Collagen Laminates
Source: Biomedicines. 2021 Nov 11;9(11):1668. doi: 10.3390/biomedicines9111668 (PMC8615449; doi:10.3390/biomedicines9111668)
Supplement: Supplementary file 1 [file biomedicines-09-01668-s001.zip › Supplementary Figure S1.pdf]

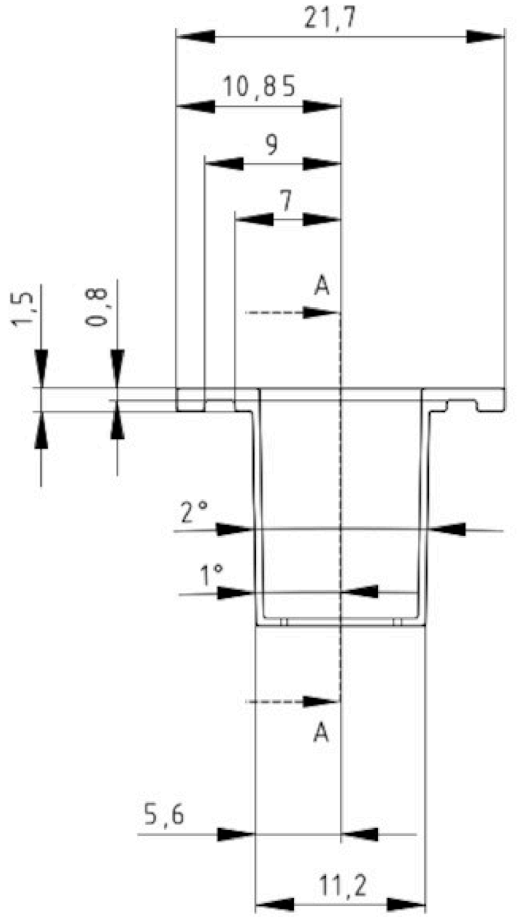

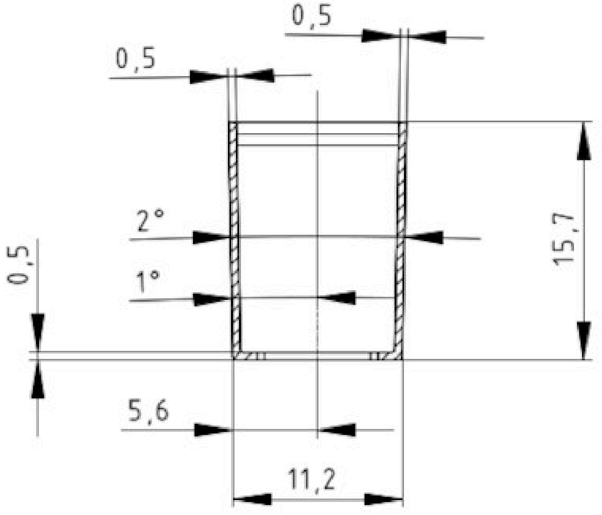

Section A - A

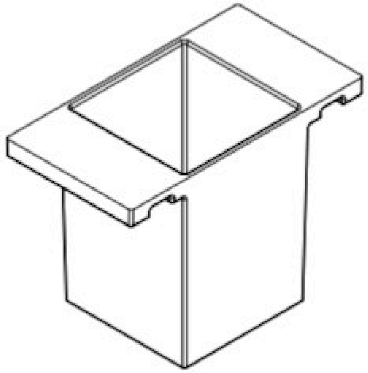

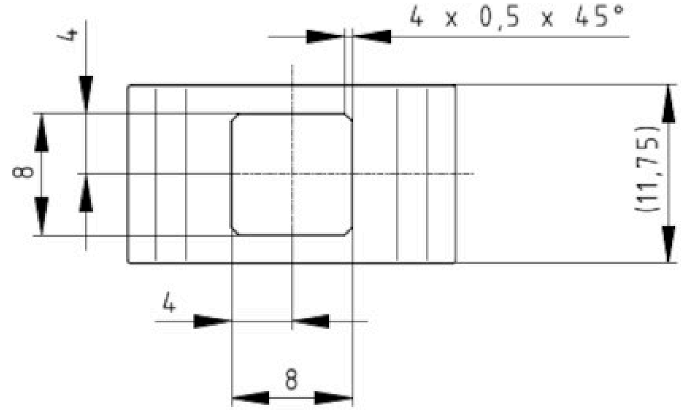

unspecified radii: 0,2 mm

DESIGNED BY: Y.M. + M.K.<br>DATE:		Lower part	
SIZE<br>A4			
SCALE<br>2:1	WEIGHT (kg)	DRAWING NUMBER	SHEET<br>1/1

This drawing is our property; it can't be reproduced or communicated without our written consent.
